# Safety and immunogenicity of Onderstepoort Biological Products’ Rift Valley fever Clone 13 vaccine in sheep and goats under field conditions in Senegal

**DOI:** 10.4102/ojvr.v82i1.857

**Published:** 2015-05-29

**Authors:** Modou M. Lo, Victor Mbao, Pascale Sierra, Yaya Thiongane, Mariame Diop, Meritxell Donadeu, Baptiste Dungu

**Affiliations:** 1 Laboratoire National de l’Elevage et de Recherches Vétérinaires, Institut Sénégalais de Recherches Agricoles, Dakar, Senegal; 2 Global Alliance for Livestock Veterinary Medicines, Malawi; 3 Independent Veterinary Consultant, Gerstheim, France; 4 Global Alliance for Livestock Veterinary Medicines, Scotland

## Abstract

This blinded field safety study was conducted in Senegal to assess safety and immunogenicity of administration of the registered dose of Rift Valley fever virus (RVFV) Clone 13 vaccine (Onderstepoort Biological Products) to sheep and goats of West African breeds under natural conditions. A total of 267 small ruminants (220 sheep, 47 goats) were included; half received RVFV Clone 13 vaccine at the recommended dose and half received the diluent (as placebo) only. The study was performed on three commercial farms in the northern and eastern region of Senegal in accordance with veterinary good clinical practices. The animals were observed daily for 3 days after vaccination, and then weekly for 1 year. In both sheep and goats vaccinated against RVFV seroconversion rates above 70% were recorded. No seroconversion related to RVFV was observed in placebo-treated animals. No statistically significant differences were determined between placebo and vaccinated groups for mean rectal temperatures for the first 3 days after administration (*p* > 0.05). No abnormal clinical signs related to treatment were noted, and only one slight injection site reaction was observed in one vaccinated animal for 2 days after vaccination. Out of 176 births assessed over 1 year (93 from the vaccinated group, 83 from the placebo group), 9 were abnormal in the placebo group and 3 in the vaccinated group (*p* > 0.05). The frequency of adverse events was similar in the placebo and vaccinated groups. RVFV Clone 13 vaccine administered according to the manufacturer's instructions was safe and well tolerated in West African breeds of sheep and goats, including animals of approximately 6 months of age and pregnant females, under field conditions in Senegal. Antibody levels persisted up to 1 year after vaccination.

## Introduction

Rift Valley fever (RVF) is an acute mosquito-borne disease affecting mainly ruminant animals and humans (Swanepoel & Coetzer 2004). It causes abortions and high mortality in young animals (Bird & Nichol 2012; Ikegami & Makino 2011). In humans it causes a severe influenza-like disease, with occasionally more serious central nervous system complications and death (Swanepoel & Coetzer 2004). RVF is caused by a single-stranded ribonucleic acid virus of the *Phlebovirus* genus in the family Bunyaviridae (Pepin *et al.* 2010). Major epidemics have occurred in many African countries, including Senegal, where the virus is believed to be endemic (Chevalier, Thiongane & Lancelot 2009).

The control of RVF has essentially relied on vaccination, using the live attenuated Smithburn vaccine or the inactivated vaccine (Smithburn 1949; Swanepoel & Coetzer 2004). Whilst these currently available RVF vaccines have been used extensively for the control of the disease, they have several shortcomings, such as poor immunogenicity, or toxicity when used in pregnant animals (Coetzer 1982; Dungu, Donadeu & Bouloy 2013). This has encouraged many research groups to develop new vaccine candidates that may address a large number of the current challenges and be suitable for use in currently RVF-free regions as well as being included in different contingency and emergency- preparedness strategies.

A number of research groups have been working on an alternative vaccine, the RVFV Clone 13 vaccine, which has been developed, evaluated (Dungu *et al.* 2010; Muller *et al.* 1995) and registered for use in cattle, sheep and goats in South Africa. The vaccine is based on a natural RVF virus mutant that carries a large in-frame deletion in the NSs gene. Evidence so far indicates that the vaccine is highly immunogenic and does not cause abortion or foetal teratogenicity in ewes and goats vaccinated during pregnancy. The risk of reversion to virulence is believed to be negligible given the substantial genetic deletion present in the vaccine virus (Muller *et al.* 1995).

Despite the occurrence of a number of RVF outbreaks in West Africa, more specifically Senegal and Mauritania (Caminade *et al.* 2014; Ndione *et al.* 2008), vaccination has never been used for the control of the disease in this region. This is mainly due to safety concerns associated with the RVF Smithburn vaccine and challenges with vaccine availability. The RVFV Clone 13 was considered to be a safer alternative for use in the region. In order to provide confidence to livestock owners and veterinary authorities, this study was designed and was aimed at evaluating the safety and efficacy (through the evaluation of immunogenicity) of Onderstepoort Biological Products’ (OBP) Clone 13 RVFV vaccine, (Onderstepoort, Republic of South Africa) in Senegalese sheep and goat breeds under local conditions, and assessing the possible spread of virus from vaccinated to unvaccinated animals.

The OBP RVFV Clone 13 vaccine was tested in Senegal under field conditions, as it may potentially represent a safer and more effective option to protect livestock in West Africa than RVF vaccines currently being used elsewhere.

This trial was designed to demonstrate the safety and immunogenicity of OBP RVFV Clone 13 vaccine in sheep and goats when administered according to the manufacturer's recommendations, and to assess spread of virus from vaccinated to unvaccinated animals in local Senegalese goats and sheep under field conditions. The trial was conducted as a controlled, randomised and blinded study following the principles of good clinical practice as detailed by the International Cooperation on Harmonisation of Technical Requirements for Registration of Veterinary Medicinal Products guideline number nine.

## Research method and design

### Setting

The study was conducted from August 2011 to October 2012 on three commercial farms in the northern (Mpal, Thille Boubacar) and eastern (Diawara) regions of Senegal, using animals bred locally and kept under local farm conditions. The locations were favoured because of active surveillance implemented in these areas for more than 15 years that demonstrated endemicity of RVF (Thiongane *et al.* 1994).

### Animals

A total of 267 sheep (Touabir and Warale breeds) and goats (Sahelian breed) were included, as indicated in Table 1. They included 220 sheep (191 females and 29 males), and 47 goats (41 females and 6 males).

**TABLE 1:** Experimental animals.

Categories	Diawara		Mpal		Thille		Total
	Males	Females	Males	Females	Males	Females	
Goats, placebo	0	1	0	0	4	18	23
Goats, vaccinated	0	2	0	0	2	20	24
Sheep, placebo	5	40	1	26	9	29	110
Sheep, vaccinated	4	40	0	28	10	28	110
**Subtotal**	**9**	**83**	**1**	**54**	**25**	**95**	**267**

Age determination was based on incisors: the presence of lactating (milk) teeth (LT) only or the number of adult teeth (AT). LT: less than 14 months old – 68 females and 31 males; 2 AT: between 14 and approximately 20 months old (29 females and 3 males); 4 AT: between 20 and 28 months old (27 females, 0 males); 6 AT: between 28 and 36 months old (36 females, 0 males) and 8 AT: more than 36 months old (72 females, 1 male).

At the time of vaccination 123 animals were pregnant (99 sheep and 24 goats), and 53 animals (46 sheep and 7 goats) became pregnant during the study, as shown in Table 2.

**TABLE 2:** Numbers of sheep and goats that gave birth in relation to time of vaccination using Onderstepoort Biological Products Rift Valley fever virus Clone 13 vaccine.

Pregnancy status at vaccination	Goats		Sheep	
	Vaccinated	Placebo	Vaccinated	Placebo
Vaccinated during first trimester of gestation	3	3	18	21
Vaccinated during second trimester of gestation	5	5	16	13
Vaccinated during third trimester of gestation	5	3	18	13
Pregnant within 2 months of vaccination	1	3	11	8
Pregnant 2 months or more after vaccination	1	2	15	12
**Total**	**15**	**16**	**78**	**67**

All animals were identified by ear tag. Appropriate treatments were given to animals in the 2 weeks preceding vaccination or placebo injection, including antiparasitic therapy with ivermectin and clorsulon.

All animals were seronegative to RVF at the start of the study, except for one goat and one sheep (both were positive at day 0 by enzyme-linked immunosorbent assay [ELISA] but not the viral neutralisation test [VNT]).

### Vaccination

The RVFV Clone 13 vaccine manufactured by OBP was used. The batch number was 13, with an expiry date of 01 January 2012. The placebo was the diluent of the same vaccine, batch number 8593, expiry date 01 April 2014. Storage conditions of the vaccine and diluent were appropriately monitored and complied with the manufacturer's instructions.

Each animal received either vaccine or placebo, at a dose of 1 mL subcutaneously. Vaccines were injected with sterile needles and syringes that were changed for each animal.

### Study personnel

All personnel were well experienced in conducting vaccine trials, and were formally trained to good clinical practice guidelines by an independent study monitor. The independent study monitor attended the crucial study time-points, including pre-study, vaccination, observation period and end of study, and maintained regular contact with Institut Sénégalais de Recherches Agricoles's (ISRA's) team throughout the observation period. Animals were examined at least weekly by the local veterinary representative and every 2 months by the investigator from ISRA Dakar.

### Serology and virology

All serology (VNT and ELISAs for IgG and IgM) and virology (virus isolation using Vero cells and conventional polymerase chain reaction) analyses were conducted at Laboratoire National de l’Elevage et de Recherches Vétérinaires (LNERV) at ISRA, Dakar.

Sera were taken from all animals before the study commenced and then from a selection of approximately 20 animals per group per site every 2 months. The samples were analysed by virus neutralisation (Davies, Jacobsen & Sylla 1988), RVF inhibition ELISA to detect IgM (National Institute for Communicable Diseases, Sandringham, South Africa; Paweska *et al.* 2005) for all samples up to day 135 and RVF recIgG ELISA to detect IgG (National Institute for Communicable Diseases, Sandringham, South Africa; Paweska, Jansen van Vuren & Swanepoel 2007) for all samples between days 195 and 365. The ELISAs used were commercial kits manufactured by Biological Diagnostic Supplies Limited, Ayrshire, Scotland.

### Study design

This study was a randomised, blinded and placebo-controlled trial conducted under field conditions. Vaccine safety was assessed by monitoring local (injection site) and general clinical reactions, including rectal temperature, once daily for the first 3 days then at least once weekly for 1 year following vaccination or placebo injection. All abnormal health observations, medical treatments and births or abortions were recorded for the year of observation.

Blood samples were taken on the day of vaccination and afterwards every 2 months (+/− 10 days) until day 365 to assess RVF antibody levels prior to vaccination and every 2 months afterwards.

Randomisation schedules were prepared for each species (sheep or goat) and each study site; half of the animals received vaccine and the other half received placebo based on order of catch.

To maintain blinding the person who administered treatments was never present at clinical examinations and the randomisation lists and treatment record forms were kept in a secure location which was not accessible to personnel conducting the health and safety observations.

### Statistical methods

Mean rectal temperatures were calculated daily for each treatment group and compared using two-factor analysis of variance (time and treatment). The frequencies of adverse events and abnormal births were compared between groups using the Chi-square test.

## Results

### Rectal temperatures

**FIGURE 1:**
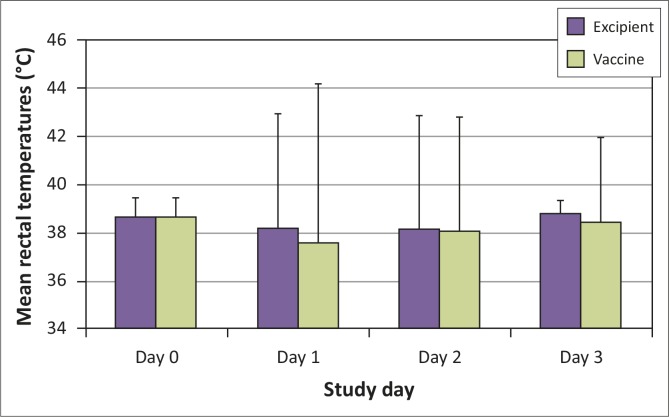
Mean and standard deviations for rectal temperature in °C from day 0 to day 3.

Mean and standard deviations for rectal temperatures from day 0 (prior to treatment) to day 3 (approximately 72 hours after treatment) are shown in Table 3 and Figure 1.

**TABLE 3:** Mean and standard deviations for rectal temperature in °C from day 0 to day 3.

Treatment	Mean RT and SD	Study day			
		Day 0	Day 1	Day 2	Day 3
Placebo	Mean RT†	38.62	38.14	38.11	38.79
	SD† RT	0.82	4.76	4.75	0.51
RVF Clone 13 vaccine	Mean RT	38.62	37.52	38.03	38.41
	SD RT	0.81	6.63	4.74	3.49

RVF, Rift Valley fever; RT, rectal temperature; SD, standard deviation.

†, rectal temperature in °C.

No statistically significant difference was noted between treatment groups for mean rectal temperature between days 0 and 3 (*p* = 0.27 or *p* > 0.05) and also no significant time x treatment interaction (*p* = 0.79 or *p* > 0.05) was recorded.

### Local tolerance: Injection site reactions

Of 267 animals that received either the placebo or the RVFV Clone 13 vaccine, only one sheep was observed to have slight swelling at the injection site on days 1 and 2. The signs disappeared after day 2 and no other abnormal signs were observed. None of the other animals presented any injection site reaction.

### General health observations

All animals were individually observed for 3 days after vaccination. During this period only one goat was recorded to have abnormal general health, diagnosed as foot rot, which was not considered to be related to the study.

In the year following vaccination or placebo injection all animals were observed weekly for abnormal health. Only those animals with health abnormalities were then individually examined. In total 45 health observations were recorded by the veterinarian (veterinary reports) in the placebo group and 50 in the vaccine group, as well as 12 potential adverse events (6 in each group). The frequency of veterinary reports was not significantly different between treatment groups (Chi-square test, *p* > 0.05). No abnormal health observations were related to the trial.

### Births and abortions

A total of 176 births were observed during the 1-year study period, 31 in does and 145 in ewes. Ninety-three births occurred in the vaccinated group and 83 in the placebo group, and of these 12 were considered abnormal (3 in the vaccine group and 9 in the placebo group). Two abnormal births were noted less than a week after the start of the trial, one in each treatment group. All other abnormal births occurred at least one month (one animal) but generally more than 2 months after injection. The offspring from the ewe that aborted 5 days after vaccination was tested for RVF by polymerase chain reaction and isolation in Vero cells, and the results were negative. The abortion was considered not to be related to vaccination. The frequency of abnormal births was not significantly different between the vaccinated group and the placebo group (Chi-square test with Yate's correction, *p* > 0.05).

### Seroconversion in the placebo group

No seroconversion to RVFV was observed in any animal from the placebo group, which supports the concept of no passage of the RVFV vaccine strain from the vaccinated animals to the non-vaccinated animals.

Natural infection did not occur, as demonstrated by lack of clinical signs and or seroconversion of the non-vaccinated animals, as well as lack of clinical signs in of neighbouring herds.

### Seroconversion after vaccination

On day 0 one goat and one sheep from the vaccinated group were positive on ELISA to IgM (RVF inhibition ELISA), and no animal was positive by VNT. In the placebo group the frequency of animals which were seropositive, both by ELISA (testing for IgM until day 135, and for IgG afterwards) and VNT stayed low throughout the observation period (as reflected in Tables 4 and 5). Amongst the animals that regularly had blood sampled, only one tested positive by VNT on consecutive samplings on days 195, 260 and 315, but remained negative on ELISA, except on day 195. This animal was then sold. Two other animals had antibody titres on VNT of 160 at one or more time-points, but became seronegative again at the following time-point. These animals were not positive on ELISA at these time-points. One animal was seropositive on day 365 only, on testing both by VNT and by ELISA.

**TABLE 4:** Enzyme-linked immunosorbent assay and viral neutralisation test results in goats.

Categories	Day 0	Day 60	Day 120	Day 195	Day 260	Day 315	Day 365
Number of vaccinated goats sampled	24	11	11	11	6	8	7
Goats positive ELISA	1	5	4	7	4	7	6
% goats positive on ELISA	4.17	45.45	36.36	63.64	66.67	87.50	85.71
Goats positive on VNT 160	0	4	4	6	4	7	5
Goats doubtful on VNT 80	0	2	4	2	1	1	2
% Goats ≥ 80 VNT	0.00	54.55	72.73	72.73	83.33	100.00	100.00
**Number placebo goats sampled**	22	6	4	5	5	6	7
Goats positive ELISA	0	0	0	0	0	0	1
% goats positive ELISA	0.00	0.00	0.00	0.00	0.00	0.00	14.29
Goats positive VNT 160	0	0	0	0	0	0	1
Goats doubtful VNT 80	0	0	0	0	0	0	0
% Goats ≥ 80 VNT	0.00	0.00	0.00	0.00	0.00	0.00	14.29

ELISA, enzyme-linked immunosorbent assay; VNT, viral neutralisation test.

**TABLE 5:** Enzyme-linked immunosorbent assay and viral neutralisation test results in sheep.

Categories	Day 0	Day 60	Day 120	Day 195	Day 260	Day 315	Day 365
**Number vaccinated sheep sampled**	110	64	61	58	58	58	55
Sheep positive ELISA	1	31	17	25	6	27	22
% sheep positive ELISA	0.91	48.44	27.87	43.10	10.34	46.55	40.00
Sheep positive VNT 160	0	35	23	31	38	26	30
Sheep doubtful VNT 80	0	12	10	10	3	6	9
% sheep ≥ 80 VNT	0.00	73.44	54.10	70.69	70.69	55.17	70.91
**Number placebo sheep sampled**	109	62	48	58	52	44	55
Sheep positive ELISA	0	1	1	5	1	0	0
% sheep positive ELISA	0.00	1.61	2.08	8.62	1.92	0.00	0.00
Sheep positive VNT 160	0	1	1	1	0	0	0
Sheep doubtful VNT 80	0	0	0	1	2	0	1
% sheep ≥ 80 VNT	0.00	1.61	2.08	3.45	3.85	0.00	1.82

ELISA, enzyme-linked immunosorbent assay; VNT, viral neutralisation test.

In the vaccine group seroconversion was observed by ELISA and VNT in more than 70% of the animals, starting on day 60. The seropositivity rates remained high throughout the observation period, including on the last study day, when more than 70% of the sheep and goats had neutralising antibody titres above the positivity threshold. Neutralising antibody titre results were generally more consistent over time for the same animal. Many animals in the vaccinated group regularly had an ELISA titre very close to but lower than the test threshold and were therefore not counted as positive. However, they definitely presented a significant rise in antibody titres over time.

The percentage of ELISA-positive animals at day 60 was as expected, based on the authors’ previous experience.

## Ethical considerations

The study protocols were reviewed and approved by the scientific committees of the Global Alliance for Livestock Veterinary Medicines and the ISRA of Dakar. The study did not involve killing, administration of pain-causing substances or invasive procedures in animals. Only the registered vaccine was used and animals were routinely bled according to a standard operating procedure. Permission to conduct the study was granted by the Ministry of Livestock in Senegal.

## Discussion

Vaccination is the most effective tool to control RVF, accompanied by other measures such as effective surveillance, a good diagnostic strategy and reliable emergency-preparedness (Dungu *et al.* 2013). Preventing RVFV infection of livestock by vaccination is a key element in breaking the chain of human epidemics, and could lead to control of this significant public health threat (Bird & Nichol 2012). A human RVF vaccine is currently not commercially available.

The vaccine used in this trial, OBP RVFV Clone 13, has been registered and used in South Africa. It is based on an avirulent RVF virus isolated from a non-fatal case of RVF in the Central African Republic that had been passaged in mice and Vero cells and then plaque purified in order to study the homogeneity of virus subpopulations (Muller *et al.* 1995). A Clone designated 13 did not react with specific monoclonal antibodies against NSs, and when further investigated was found to be avirulent in mice, yet immunogenic (Muller *et al.* 1995; Vialat *et al.* 2000). This vaccine has been evaluated for safety and efficacy in sheep and cattle (Dungu *et al.* 2010; Von Teichman *et al.* 2011).

Unlike the Smithburn and MP-12 strains of RVFV vaccines, RVFV Clone 13 vaccine does not appear to induce abortions in pregnant ewes, making it one of the most promising live attenuated vaccine candidates thus far (Boshra *et al.* 2011). Results of experiments in sheep demonstrated that RVFV Clone 13 vaccine also has a better safety profile than other RVFV mutants, such as MP-12 and Smithburn (Dungu *et al.* 2010). The safety and efficacy of RVFV Clone 13 vaccine in cattle and sheep under the recommended conditions of use are also supported by the administration of more than 10 million doses during the 2009–2010 RVF outbreak in South Africa, where the vaccine played a key role in the control of the disease (Dungu *et al.* 2013). This vaccine has the advantage over inactivated vaccines as it is more cost-effective, requiring only one vaccination instead of two administrations, and can be produced in larger batches than the inactivated vaccine (Dungu *et al.* 2010).

The RVFV Clone 13 vaccine has not been used in West Africa, despite the presence of RVF in the region (Chevalier et  al. 2009; Clements *et al.* 2007). Prior to the field evaluation described here, this vaccine was tested in West African goats and sheep under laboratory conditions at ISRA Dakar (data not shown) as well as in field conditions in Kenya. In both studies the vaccine proved to be safe and induced a high level of seroconversion (B. Dungu, pers. comm., January 2012).

This is the first report on safety and immunogenicity data for the OBP RVFV Clone 13 vaccine in goats and sheep under field conditions in Senegal. The results are very encouraging, as the vaccine was confirmed to be safe in local breeds of both species in West Africa. Efficacy, evaluated as seroconversion after vaccination, was observed in more than 70% of the animals, which should ensure protective immunity at herd level. In experimental studies very good neutralising antibody responses measured by the World Organisation for Animal Health-recommended VNT were recorded in all RVFV OBP Clone 13 vaccinated sheep from day 7, and in some sheep up to day 60 post-vaccination (Dungu *et al.* 2010). In addition, in this field trial no signs of toxicity resulting from vaccination were detected when it was used in pregnant animals.

In conclusion, a total of 267 sheep and goats of local breeds were included in this field study to evaluate the safety and immunogenicity of the administration of the registered dose of RVFV Clone 13 vaccine. The animals were kept under local conditions and observed for 1 year after vaccination or placebo injection. More than 70% of the sheep and goats vaccinated with the OBP RVFV Clone 13 vaccine showed long-term seroconversion that persisted for the 1 year during which the trial was conducted. No signs of local or general intolerance and no signs of toxicity to vaccination of pregnant ewes and goats were noted. Vaccination with RVFV Clone 13 vaccine was safe and well tolerated in sheep and goats of Senegalese breeds, including pregnant females, under field conditions. These results suggest that vaccination of small ruminants with this vaccine could be effective in controlling RVF.
